# The Effect of Meditation on Comprehension of Statements About One-Self and Others: A Pilot ERP and Behavioral Study

**DOI:** 10.3389/fnhum.2019.00437

**Published:** 2020-01-09

**Authors:** Alexander Savostyanov, Sergey Tamozhnikov, Andrey Bocharov, Alexander Saprygin, Yuriy Matushkin, Sergey Lashin, Galina Kolpakova, Klimenty Sudobin, Gennady Knyazev

**Affiliations:** ^1^Laboratory of Psychological Genetics, Institute of Cytology and Genetics of Siberian Branch of the Russian Academy of Sciences (SB RAS), Novosibirsk, Russia; ^2^Laboratory Differential Psychophysiology, State-Research Institute of Physiology and Basic Medicine, Novosibirsk, Russia; ^3^Laboratory of Biological Markers of Human Social Behavior, Humanitarian Institution at the Novosibirsk State University, Novosibirsk, Russia

**Keywords:** Samatha meditation, assessment of self and other, recognition of written speech, EEG and ERP, task performance time and accuracy of error detection

## Abstract

The main goal of this study was to examine the effect of long-term meditation practice on behavioral indicators and ERP peak characteristics during an error-recognition task, where participants were presented with emotionally negative (evoking anxiety or aggression) written sentences describing self-related or non-self-related emotional state and personality traits. In total, 200 sentences written in Russian with varying emotional coloring were presented during the task, with half of the sentences containing a grammatical error that the participants were asked to identify. The EEG was recorded in age-matched control individuals (*n* = 17) and two groups of Samatha meditators with relatively short- (3–5 years’ experience, *n* = 18) and long-term (10–30 years’ experience, *n* = 18) practice experience. Task performance time (TPT) and accuracy of error detection (AED) were chosen as behavioral values. Amplitude, time latency and cortical distribution of P300 and P600 peaks of ERP were used as a value of speech-related brain activity. All statistical effects of meditation were estimated, controlling for age and sex. No behavioral differences between two groups of meditators were found. General TPT was shorter for both groups of meditators compared to the control group. Non-meditators reacted significantly slower to sentences about aggression than to sentences about anxiety or non-emotional sentences, whereas no significance was found between meditator groups. Non-meditators had better AED for the sentences about one-self than for the sentences about other people, whereas the meditators did not show any significant difference. The amplitude of P300 peak in frontal and left temporal scalp regions was higher for long-term meditators in comparison with both intermediate and control groups. The latency of P300 and P600 in left frontal and temporal regions positively correlated with TPT, whereas the amplitude of P300 in these regions had a negative correlation with TPT. We demonstrate that long-term meditation practice increases the ability of an individual to process negative emotional stimuli. The differences in behavioral reactions after onset of negative information that was self-related and non-self-related, which is typical for non-meditators, disappeared due to the influence of meditation. ERP results could be interpreted as a value of increase in voluntary control over emotional state during meditational practice.

## Introduction

Assessment of self and others (non-self) is one of the most important elements of regulation of social behavior. One of these methods, attribution theory, was suggested by social psychologist Heider (1958) to evaluate how people perceive their own behavior, as well as the behaviors of others. According to this theory, an act of attribution is a prediction of ongoing behaviors, such as emotions and motives for self or any other person. The main question of research in this area of self-feeling (our “I”) is whether it has a special status in human consciousness or if it is functionally equivalent to semantic processing of any other classes of stimuli. Is our consciousness capable of paying more attention to information in which we are somehow involved? Symons and Johnson (1997) showed that information related to self in any way is remembered better than impersonal. This phenomenon became known as “self-reference effect”. Neuroscientific studies on self-concept have mainly relied on experimental paradigms in which participants evaluated sentences that described some traits about the self. These studies revealed consistent activation in a medial prefrontal cortex (mPFC) during self-evaluation (see meta-analyses: Denny et al., 2012; Murray et al., 2012). In the study of Fossati et al. (2003), influence of emotional valence of stimuli was studied in addition to localization of the information processing relating to self. Activation in the right dorsomedial prefrontal cortex was found for self-referencing stimuli, which was independent from the valence of the words. Differences in processing of positive and negative adjectives were noticed in this study in areas outside of the medial prefrontal cortex. Other studies (Moran et al., 2006; Qin and Northoff, 2011) showed that besides mPFC, such areas as the cingulate cortex, precuneus, and temporal-parietal cortex are also involved in the processing of information about self.

One of the experimental approaches to studying evaluation of self and non-self is a comparison of groups of people that vary significantly in behavioral values related to social perception. Such groups can include people of different age (Pfeifer et al., 2009), gender (Cross and Madson, 1997), or ethno-cultural traits (Markus and Kitayama, 1991; Cross et al., 2011) that affect self-evaluation. In addition, healthy subjects can be compared to psychiatric patients with impaired self-perception processes (Sass and Parnas, 2003; Nelson et al., 2012; Mishara et al., 2014). In this study, we aimed to examine people practicing Buddhist Samatha meditation through neuroscientific approaches.

Meditation is a religious practice of great interest to neuroscience, as it is a model that allows the studying of changes occurring in regards to brain function, which occur due to voluntary and conscious efforts of the subject. Meditation’s effects on attention have been previously studied using attention network task (ANT) and other experimental paradigms (Chiesa et al., 2011). Some studies report improvement in conflict monitoring (Wenk-Sormaz, 2005; Chan and Woollacott, 2007; Slagter et al., 2007; Tang et al., 2007; Moore and Malinowski, 2009). For example, it is shown that several days (20 min each day) of integrative mental-body training led to improved conflict monitoring (Tang et al., 2007). Additionally, longitudinal studies of 3-month long awareness meditation have shown decrease in attention blinking due to exercise (Slagter et al., 2007; van Leeuwen et al., 2009). In cross-section studies, experienced meditators demonstrated the best results in monitoring conflicts (van den Hurk et al., 2010).

According to the report of Aftanas and Golosheykin (2005), Sahaja Yoga meditators manifested smaller emotional arousal while watching aversive video clips, which was reflected in changes of spectral power in their EEG records. In addition, meditators manifested larger power values in theta-1 (4–6 Hz), theta-2 (6–8 Hz) and alpha-1 (8–10 Hz) frequency bands compared to control subjects in eye-closed conditions without external stimulation. These results were interpreted as proof of meditators having better capabilities to moderate intensity of emotional arousal.

Changes in amplitude of N1, P2, and P3 peaks in meditators compared to non-meditators were revealed in many ERP studies of different kinds of meditation (Cahn and Polich, 2009; Atchley et al., 2016; Biedermann et al., 2016). P300 amplitude increased in meditators immediately after meditation practice compared to the pre-meditation condition (Telles et al., 2019). The study of Vipassana meditation practice has found that the dynamics of P300 in conjunction with the theta and alpha bands spectral power indices reflected differences between people with different prolongation of meditation experience (Kakumanu et al., 2019). The amplitude of frontal P300 reflects a degree of concentration of voluntary attention. Therefore, most researchers interpret the influence of meditation on the amplitude of P300 as an index of the increase in the ability of meditators to voluntary concentrate attention on themselves and/or external events.

Several functional and structural MRI studies of awareness meditation have focused on neuroplasticity in areas responsible for attention control. Awareness practice effects on attention are mostly related to the anterior cingulate (van Veen and Carter, 2002; Cahn and Polich, 2006; Hölzel et al., 2007; Tang et al., 2010, 2012a,b, 2013; MacCoon et al., 2014; Tang and Posner, 2014). The anterior cingulate is responsible for executive attention and control through detecting conflicts caused by incompatible information processing flows (van Veen and Carter, 2002; Posner et al., 2007; Tang and Tang, 2014). The anterior cingulate cortex and the frontal cortex form a part of the network, which, due to effective distant connections with other areas of the brain, facilitates cognitive processing (Sridharan et al., 2008; Tang et al., 2012b). Compared to the control group, experienced practitioners have shown an increase in activation of areas of the anterior cingulate during meditation (Hölzel et al., 2007) or with a conscious expectation of pain stimulus (Gard et al., 2012). In a controlled randomized longitudinal study, high activation of the ventral and/or rostral anterior cingulate cortex have also been identified during the rest period following 5 days of integrative mental-body training (MacCoon et al., 2014). Activation of the anterior cingulate cortex may increase during the initial stages of the practice and decrease with prolonged training (Brefczynski-Lewis et al., 2007).

Other brain areas related to attention, in which functional changes due to awareness meditation were witnessed, include the dorsolateral prefrontal cortex (dlPFC), where an increased response during executive processing was observed (Allen et al., 2012), and the posterior parietal cortex (PPC), which had shown higher activation after the MBSR course in subjects with social anxiety (Goldin and Gross, 2010).

Thus, the areas of the brain where activity changes under the influence of meditation and the areas involved in the self-assessment and evaluation of other people overlap to a significant degree. According to the self-report of participants, Samatha is a religious practice, with the end goal being the “dissolution” of Myself in Universe. Self-awareness is considered by adherents of such meditation an illusion that must be discarded. We have proposed that the long practice of Samatha can change the processes of evaluation of one-self and/or of other people, which can be witnessed using brain electrical activity analysis. We chose event-related potentials (ERP), occurring during the recognition of written sentences, which include emotionally negative descriptions of one-self’s conditions or non-self traits and characteristics, as the method of our study. According to our hypothesis, in such a paradigm non-meditators should show differences in responses to messages about self and non-self, whereas the consequence of meditation should be the disappearance of such differences.

## Materials and Methods

### Participants

The study was conducted at the Baikal Meditational Center^1^. The experimental sample included 53 healthy right-handed participants from 25 to 66 years (32 men; mean age = 41.0, *SD* = 8.3). The participants were divided into three groups: (1) non-meditators-17 persons (10 men, mean age = 40.5, *SD* = 8.5) who refused to take part in a meditational practice; (2) intermediate group–18 persons (11 men, mean age = 40.3, *SD* = 8.0) with 3–5 years of experience of meditation; and (3) long-term meditators–18 persons (11 men, mean age = 42.7, *SD* = 9.3) who had long-term (more than 10 years) experience of a meditation practice. All participants from the long-term group have the status of great masters of meditation, recognized in the Buddhists community. After completion of our study, they took part in “a retreat,” i.e., semi-annual continuous meditation. The groups of participants were balanced in age and sex. All participants had no history of neurological, psychiatric, or major somatic disorders. According to the self-report, they denied use of narcotic drugs or other psychoactive substances. All participants were native Russian-speakers or natural bilinguals (Russian and one of the Siberian languages) and had normal or corrected-to-normal visual acuity. All participants (including non-meditators) are related to the lamaistic Buddhism community of Russia.

After the experiment all participants filled a Russian version of Goldberg’s IPIP Big-Five Factor Markers (validated by Knyazev et al., 2010) for estimation of their psychological traits. In addition, a set of psychological questionnaires was used for estimation of participants’ differences in emotional intelligence scores, trait anxiety level, vulnerability to depression and anxiety disorder, etc.

All subject protection guidelines and regulations were followed in accordance with the Declaration of Helsinki. The study goals were explained to all participants and they signed the informed consent. The study and the consent form were approved by the Institute of Physiology and Basic Medicine ethics committee and by the spiritual leader of the lamaistic Buddhism community of Russia.

### Experimental Procedure

Two-hundred sentences in Russian language were selected for the experiment (see Table 1). Half of the sentences (100) contained a grammatical error. In preliminary testing on another group, the grammatically wrong sentences were selected so that they were easily recognized (more than 80% accuracy) by all participants. The text stimuli were presented in white and black (Arial, 36 pt) *via* a 24.4 cm × 18.3 cm monitor located 60 cm away in front of a participant. A warning signal (cross) appeared at the center of the screen for 0.5 s before the task onset. The participants were instructed to answer whether a presented sample contains an error by pressing one of two buttons with their dominant hand. Participants had three practice trials before task execution. Time for decision making was not limited for participants, but they were instructed to perform the task as quickly as possible and with maximal accuracy.

**Table 1:** Examples of sentences from the different categories.

Category of sentences	Right sentences	Wrong sentences
My aggression	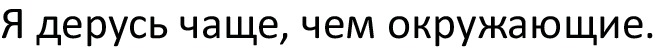	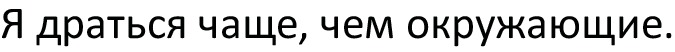
Aggression of others	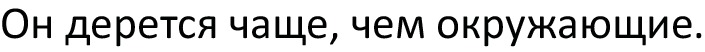	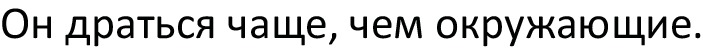
My anxiety	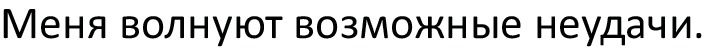	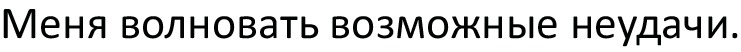
Anxiety of others	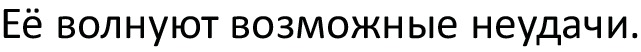	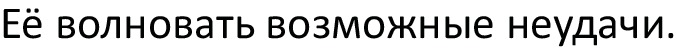
Inanimate objects	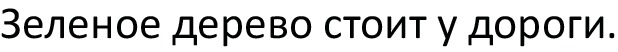	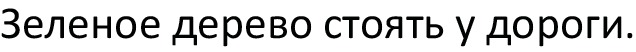

The experiment also contained a hidden condition: the sentences were grouped by emotional coloring, of which participants were not informed. There were five groups of sentences: (1) sentences describing aggression of a participant; (2) sentences describing aggression of other people; (3) sentences describing anxiety of a participant; (4) sentences describing anxiety of other people; and (5) neutral sentences about inanimate objects. The sentences from different categories were presented in random order. The sentences about anxiety were selected from the Russian version of Spielberger’s State-Trait Anxiety Inventory (Spielberger et al., 1970; translated to Russian and validated by Hanin, 1976). The sentences about aggression were taken from the Buss-Perry aggression questionnaire (Buss and Perry, 1992; translated to Russian and validated by Yenikolopov and Tsibulsky, 2007). All sentences about one-self were taken from validated Russian versions of psychological tests. Translations of these tests into Russian were performed by professional translators. All questionnaires were repeatedly validated in different samples in Russia and in other countries where Russian is widely spoken. The sentences about non-self anxiety or aggression were created by the replacement of a pronoun “I” or “Me” on accidentally chosen pronouns “He,” “She,” “They,” “Him,” “Her” or “Them.” The sentences about objects, anxiety, and aggression were balanced in number of words and grammatical structure. The sentences about self and non-self differed only by pronouns and connected verbs. The order of sentences was randomized across participants.

### EEG Recording

EEGs were recorded using 130 channels (128 EEG, VEOG, ECG) *via* Ag/AgCl electrodes. The EEG electrodes were placed on 128 head sites according to the extended International 5–10% system and referred to Cz with ground at FzA. The Quik-Cap128 NSL was used for electrode fixation. The electrode resistance was maintained below 5 kΩ. The signals were amplified using NVX 136 amplifier (MCS, Russia), with 0.1–100 Hz analog bandpass and continuously digitized at 1,000 Hz.

### Behavioral Data Processing

The task performance time (TPT, in milliseconds) and accuracy of error detection (AED, a percent of correctly recognized error in wrong sentences and a percent of sentences without error correctly marked as “right”) were used as the two behavioral values.

Initially, these values were used for repeated measures ANOVA with the Greenhouse-Geisser correction to test the main effects of such factors as “group” (non-meditators vs. meditators), “correctness” (right or wrong sentences), “sentence category” (five levels for different categories of sentences), “me_other” (sentences about participant or about other people, results for neutral sentences were excluded from such analysis), “aggression_anxiety” (sentences about aggression vs. about anxiety, results for neutral sentences were excluded from such analysis), age, sex, and interactions between these factors. However, any significant differences in the behavioral values between the participants from intermediate and long-term experience groups were not revealed. Both groups of meditators showed same differences from the non-meditators. For this reason, behavioral values were compared between the united group of meditators and the control group of non-meditators. Besides, no inter-group differences in TPT and AED were found for neutral sentences. Therefore, behavioral values for neutral sentences were excluded from statistical analysis. Finally, for statistical comparison of TPT and AED values, the repeated measures ANOVA with the Greenhouse-Geisser correction with factors “group” (non-meditators vs. meditators of both groups), “correctness,” “me_other,” “aggression_anxiety,” age, sex, and interactions between these factors was used. All statistical effects of meditation were estimated controlling for age and sex.

### EEG Pre-processing and ERP Analysis

Recordings were processed in the EEGLAB toolbox (Delorme and Makeig, 2004). The trials containing artifacts were rejected from analysis. For each subject, 180–195 trials with sentence onset were used. The time intervals from 1.5 s before to +3.0 s after the fixation cross onset were analyzed. Time intervals from −1.5 to −0.75 s before fixation cross onset were used for baseline-correction.

EEGs were preliminary band-pass filtered in 1–40 Hz using elliptic filters. Following the suggestion by Delorme and Makeig (2004), re-reference (averaged reference) and baseline adjustment procedures were performed during data pre-processing. Independent component analysis (ICA) was used for correction of eye-movement and eye-blinking artifacts. First, the component’s weights were computed individually for each record. Then, components corresponding to eye’s artifacts were disclosed by visual inspection of component sets together with VEOG and ECG records. Artifactual components were removed in the pre-processing of EEGs.

To assess changes in signal amplitude, associated sentence onset, event-related potentials (ERPs) were calculated in the ERPLAB toolbox^2^. After removing artifacts, we computed ER*P*-values using the Erplab toolbox separately for every EEG-channel, subject and experimental condition, and cutoff filter at 12 Hz was applied to them. After that, maximal peak amplitudes, mean peak amplitude, and peak latencies in 150–500 ms (that corresponded to location of P300 peak during the visual peak analysis) and 600–1,000 ms (i.e., peak P600) time ranges were averaged across nine scalp regions of interest (ROI: left frontal, medial frontal, right frontal, left temporal, central, right temporal, and left, medial and right parietal-occipital scalp regions) for each subject. These values were used for repeated measures ANOVA with the Greenhouse-Geisser correction to test the main effects of such factors as “region of interest,” “group,” “correctness,” “sentence category,” age, sex, and interactions between these factors.

## Results

### Behavioral Results

No statistically significant inter-group differences in Big-Five factor markers were found in all scales of Goldberg’s inventory. The main effects of factors “age” and “sex” and their interactions were statistically insignificant both for TPT (for age *p* = 0.95, for sex *p* = 0.51) and AED (for age *p* = 0.33, for sex *p* = 0.12).

As already mentioned, significant differences in the behavioral values between the participants from intermediate and long-term experience groups were not revealed. The main effect of “correctness” factor controlling for age and sex was highly significant as for TPT, *F*_(1,51)_ = 23.78; *p* < 0.0001, as for AED, *F*_(1,51)_ = 15.65; *p* < 0.0001. The correct sentences were recognized slower (correct sentences: mean TPT 4.0 ± 0.2 s; wrong sentences: 3.4 ± 0.2 s), but with better accuracy (correct sentences: mean accuracy 96.5 ± 1.3%; wrong sentences: 90.6 ± 0.9%), than the sentences with error. However, the interactions between factors “correctness,” “group,” “age,” and “sex” were insignificant for both values (*p* > 0.5).

**Figure 1:**
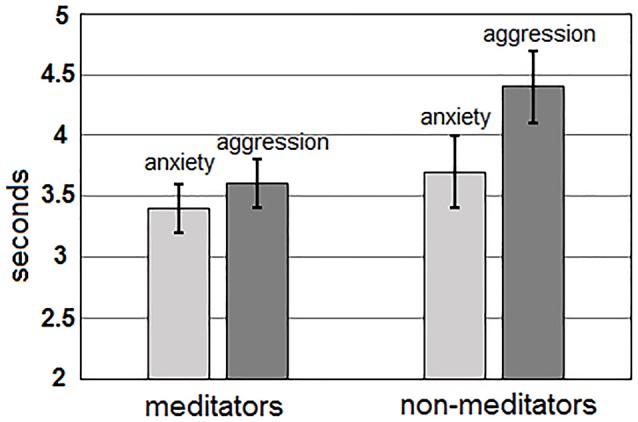
Differences in time of task performance for the sentences about anxiety (light gray) and aggression (darkly gray) for both groups of meditators (left side) and non-meditators (right side).

For TPT, the main effect of the “group” factor was statistically significant, *F*_(1,51)_ = 2.95; *p* = 0.052. Meditators solved the task faster (mean time 3.3 ± 0.2 s) than non-meditators (4.0 ± 0.2 s). In addition, for value of TPT, the main effect of the “me/other” factor was highly significant, *F*_(1,51)_ = 22.69; *p* < 0.0001. Sentences about one-self were recognized faster (mean time 3.5 ± 0.2 s), than about others (3.9 ± 0.2 s). Interaction between factors “Me/Other” and “group” for value of reaction time was statistically insignificant (*p* > 0.3). Additionally, a highly significant main effect of the “Anxiety/Aggression” factor, *F*_(1,51)_ = 51.24; *p* < 0.0001, and an interaction between “Anxiety/Aggression” and “group” factors, *F*_(1,51)_ = 5.86; *p* = 0.019 were revealed for TPT. For all groups of participants, recognition time for sentences about anxiety (mean 3.4 ± 0.2 s) was lower than for sentences about aggression (4.0 ± 0.2 s). However, meditators had smaller differences in recognition time for sentences about anxiety and aggression (anxiety: 3.4 ± 0.2 s; aggression: 3.6 ± 0.2 s) than non-meditators (3.7 ± 0.3 s 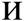 4.4 ± 0.3 s, respectively; Figure 1).

**Figure 2:**
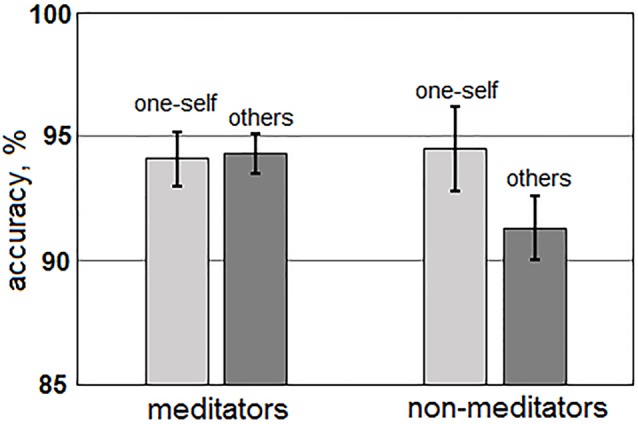
Differences in accuracy of error detection (AED) for the sentences about one-self (light gray) and others (darkly gray) for both groups of meditators (left side) and non-meditators (right side).

The main effect of the “group” factor for AED value was not significant (*p* > 0.4). For AED, a statistically significant main effect of the “me/other” factor, *F*_(1,51)_ = 7.46; *p* = 0.009, and an interaction between “Me/Other” and “group” factors, *F*_(1,51)_ = 9.11; *p* = 0.004 were revealed. Averages between all group AED values were higher for sentences about one-self (94.3 ± 1.0%), than about others (92.8 ± 0.8%). However, these differences manifested only in the non-meditation group (one-self: 94.5 ± 1.7%; other: 91.3 ± 1.3%), while meditators had no such differences (one-self: 94.1 ± 1.1%; others: 94.3 ± 0.8%; Figure 2). The main effect of the “Anxiety/Aggression” factor (*p* > 0.9), and the interaction between “Anxiety/Aggression” and “group” (*p* > 0.8) were not significant. Interactions between “Me/Other,” “Anxiety/Aggression,” “age,” and “sex” were also insignificant.

### ERP Results

**Figure 3:**
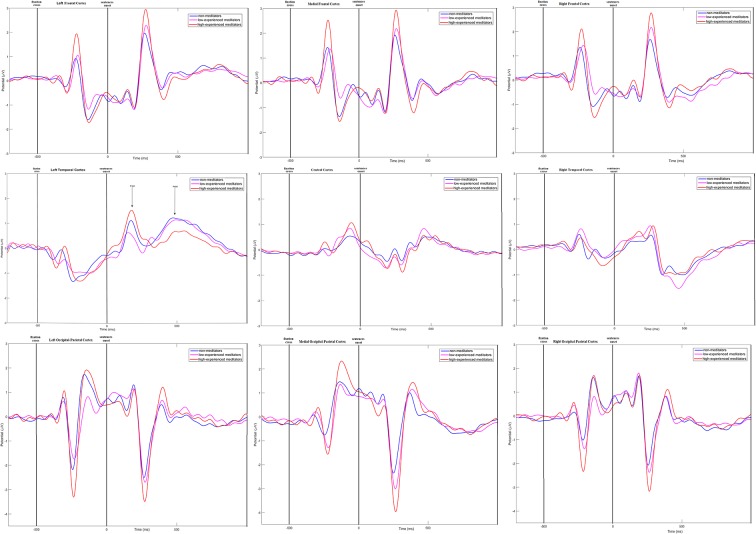
Time-amplitude plots of ERP in nine cortical ROI for three groups of participants. Blue line—ERP for control group; pink line—ERP for short-term meditators; orange line—ERP for long-term meditators. First vertical line notices a moment of fixation cross onset; second vertical line notices a moment of sentence onset.

**Figure 4:**
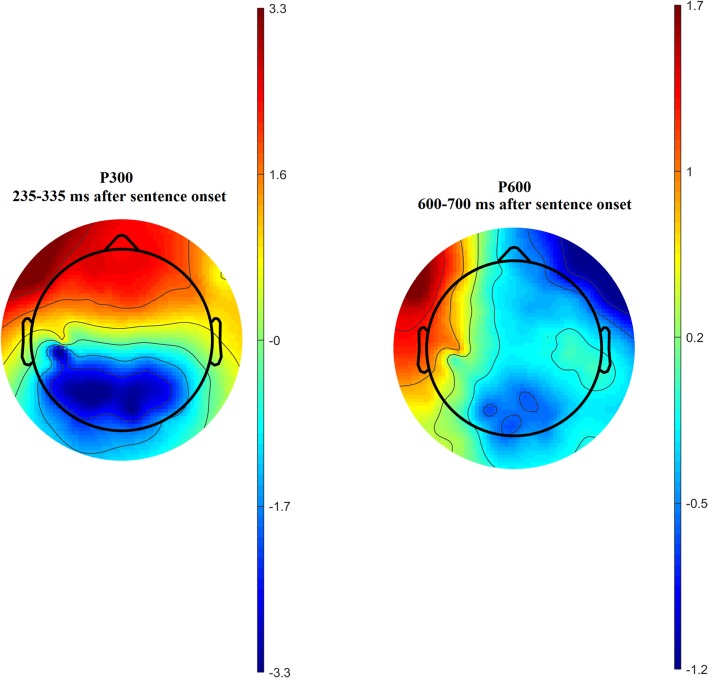
Cortical distribution of P300 (left side) and P600 (right side) amplitude averaged across all participants.

Time-amplitude plots of ERP in different cortical areas among different groups of participants are presented in Figure 3; cortical topography is presented in Figure 4. P300 and P600 peaks can be identified by visual inspection of these plots. P300 peak is clearly noticeable in the left temporal and all frontal cortical regions with amplitude maximum about 300 ms after sentence onset. Negative peak is detected in occipital-parietal cortical regions with amplitude maximum about 350 ms after sentences onset. The P300 has the highest amplitude in the group of long-term meditators in comparison with other groups. P600 peak is noticeable only in left temporal regions, i.e., in Broca’s and Wernicke’s areas. Amplitude of P600 among different groups is maximal in short-term meditators, but this difference was not significant. The topographic distribution of P300 and P600 peaks over cortical regions was not principally different among groups of participants. Though, general time-amplitude parameters and cortical distribution of P300 and P600 peaks were in consistence with standard patterns for tasks on language recognition.

In all subject groups, amplitudes of P300 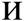 P600 peaks were significantly higher for sentences about aggression than for sentences about anxiety and inanimate objects. Amplitudes of these peaks were higher for incorrect sentences, however, no significant interactions between “group” and “category” or “correctness” were revealed. Also, there were no differences between ERP values for sentences about self and non-self. That’s why we averaged ERP values for all sentence categories.

For the majority of measured ERP values (i.e., time latency of P300 and P600, averaged and maximal amplitude of P600, and averaged amplitude of P300) any inter-group differences were not revealed. Significant inter-group differences were revealed for the maximal amplitude of the P300 peak. The main effect of age was significant, *F*_(1,46)_ = 6.16; *p* = 0.017 while the main effect of sex was insignificant (*p* > 0.7). The statistical significance of effects of meditation to P300 amplitude controlling for age and sex was higher, than without such control. The main effect of the “group” factor for P300 values was insignificant (*p* > 0.2). However, the interaction of effects of factors “Region” and “Group” compared in three groups of participants was statistically significant, *F*_(16,368)_ = 1.83; *p* = 0.026. Using one-way ANOVA, we have compared all pair combinations of three groups (i.e., control vs. intermediate; control vs. long-term meditators; intermediate vs. long-term). The comparison was performed separately for each cortical region, controlling for age and sex. One-way ANOVA revealed significant differences between the long-term meditators and other participants in left frontal, medial frontal, right frontal, and left temporal cortical regions. In all of these regions, long-term meditators showed higher amplitude of peak P300 in comparison with non-meditators and people with short-term experience of meditation (see Table 2). These effects were significant both for comparison between control participants and long-term meditators, and between short-term and long-term meditators. The significant differences between a control group and short-term meditators were revealed in right frontal and right temporal cortical regions. The amplitude of P300 in these regions was higher in control participants than in short-term meditators.

**Table 2:** The differences in the P300 maximal amplitude between various experimental groups among cortical regions.

Region of interest	Non-meditators (group 1)	Intermediate meditators (group 2)	Long-term meditators (group 3)	*P*-level between 1 and 2 groups	*P*-level between between 1 and 3 groups	*P*-level between between 2 and 3 groups
Left frontal	**2.5 ± 0.3**	**2.2 ± 0.3**	**3.1 ± 0.3**	0.56	**0.043**	**0.039**
Medial frontal	**2.5 ± 0.3**	**2.1 ± 0.2**	**2.9 ± 0.3**	0.27	**0.049**	**0.045**
Right frontal	**2.4 ± 0.3**	**1.8 ± 0.2**	**2.9 ± 0.3**	**0.052**	**0.050**	**0.050**
Left temporal	**1.9 ± 0.3**	**1.6 ± 0.3**	**2.8 ± 0.4**	0.48	**0.013**	**0.001**
Central	1.0 ± 0.2	1.0 ± 0.1	0.9 ± 0.1	0.92	0.51	0.46
Right temporal	1.8 ± 0.2	1.2 ± 0.2	1.7 ± 0.2	**0.047**	0.41	0.24
Left parietal-occipital	1.9 ± 0.3	1.7 ± 0.2	1.9 ± 0.3	0.47	0.95	0.47
Medial parietal-occipital	2.1 ± 0.3	1.9 ± 0.2	2.1 ± 0.3	0.57	0.81	0.74
Right parietal-occipital	2.2 ± 0.3	2.1 ± 0.2	2.1 ± 0.3	0.80	0.90	0.91

*Significantly different values are marked by a bold font*.

### Correlations Between Behavioral and ERP Values

Two-tailed Pearson’s correlation coefficients were calculated between the values of TPT and AED, averaged across all categories of sentences, and values of maximal amplitude and time latency of P300 and P600 peaks of ERP separately in all cortical ROI. Maximal amplitude of P300 had significantly negative correlation with the TPT in left frontal (*r* = −0.34; *p* = 0.014) and left temporal (*r* = −0.31; *p* = 0.028) cortical regions. The latency of P300 significantly positively correlated with TPT values in left frontal (*r* = 0.34; *p* = 0.014) and medial frontal (*r* = 0.31; *p* = 0.029) cortical regions. The latency of P600 had a positive highly significant correlation with TPT in the left frontal (*r* = 0.53; *p* < 0.0001) and left temporal (*r* = 0.56; *p* < 0.0001) regions, and negative correlation with accuracy of error recognition in the left temporal area (*r* = −0.30; *p* = 0.035). Separated statistical analysis did not reveal any differences in correlations between behavioral and ERP values among different groups of participants and different categories of sentences.

### Results Summary

Short-term and long-term experience groups of meditators were not statistically different in their behavioral values, however, they showed significant differences to the control group. Non-meditators showed decrease in TPT for sentences about aggression when compared to sentences about anxiety, while both meditators groups had these differences decreased due to faster recognition of sentences containing aggression. Additionally, non-meditators performed better in sentences about self than about non-self, while meditators had no such differences due to better performance in sentences about non-self. In ERP values long-term meditators showed higher amplitudes of P300 peak in frontal and left temporal cortical regions compared to other groups, while differences between non-meditators and short-term meditators were found in the right frontal and temporal regions. For all groups and all sentences, categories peak latencies of both P300 and P600 correlated positively with TPT. P300 peak amplitude in left frontal and temporal regions correlated negatively with TPT.

## Discussion

The aim of our study was to investigate how meditation changes perception of information connected with negative assessment of one-self or other people. The task reflecting implicit perception of emotional coloring of written speech was used as an experimental method. In our experiment, the voluntary attention of a participant was concentrated on detection of a grammatical error in the presented sentence, with the tasks being simple for the participants (mean AED was more than 90% for all participants). However, we compared speeds and accuracy of the answers for different categories of sentences which differed in their emotional coloring (neutral, anxious and aggressive), and whether the sentence was in relation to themselves or to other people. In this study, we have analyzed differences in behavior that participants did not control voluntarily and did not concentrate their attention on. It is possible to assume that the recorded differences in these measurements between meditators and non-meditators reflect some long-term changes in their behavior, which can be shown in other test tasks and ordinary life.

We have found two significant effects of meditation on the behavioral values and one effect of meditation on the ERP values. The behavioral differences were revealed in both groups of meditators (i.e., both for people with relatively shorter and longer experience of such practice), whereas the ERP differences distinguished the group with long-term experience both from non-meditators and meditators with shorter experience. A slight difference in the P300 amplitude in the right frontal and right temporal areas was also found between the control participants and meditators with shorter experience.

The significant differences in time and accuracy in performing the set tasks for anxiety and aggression colored sentences, as well as for sentences about self and non-self were observed in non-meditators compared to meditators (the values for neutral sentences did not significantly differ from values for sentences about self-related anxiety). Non-meditators have recognized the sentences about anxiety quicker (difference approximately in 0.7 s) than sentences about aggression. The quality of recognition of sentences about anxiety and aggression in non-meditators did not differ. Also, non-meditators recognized the sentences about themselves quicker (difference in 0.4 s) and with better accuracy (difference about 3%), than the sentences about others. It is essential that the sentences about themselves and about others differed only with a pronoun and connected verb and contained identical grammatical errors. The order of presentation of these sentences have been randomized between participants. Thus, the differences in behavioral values for these sentences cannot be connected neither with their grammatical properties, nor with a presentation order, and reflect personal features of perception of negative information about self and others.

It is also important to note that all statistically significant effects of meditation were calculated controlling for participants’ age and sex. Thus, behavioral and ERP differences between groups cannot be explained as a random result of influence of age and sex differences between groups of participants. We revealed a general correlation between TPT and AED, which also correlated with P300 amplitudes for all groups, as noted in the results on correlations between different indicators. However, long-experienced meditators showed a general decrease in TPT as compared to other groups, but the main effect of the “group” factor for AED value was not significant. Therefore, the effect of meditation on AED cannot be explained by the decrease of reaction time. In addition, the most important difference between meditators and non-meditators was not the overall accuracy or task response time, but the difference in AED when searching for an error in self-related and non-self-related sentences. This effect did not depend on the tasks response time. Also, the statistical significance of this effect increased when controlling for age and sex. Overall, the most significant behavioral effect of meditation did not depend neither on the general response time, nor on general quality of task performance, nor sex, nor age of the participants.

It has been shown that the recognition of grammatical errors in sentences depends on their emotional coloring. Sentences with emotionally negative signals usually induce a slower error recognition response, with worse accuracy scores when compared to emotionally positive or neutral sentences (Ayusheeva et al., 2018). In our results, it can be observed that in non-meditators the greatest delay and decline in quality of task performance happens in sentences containing aggression towards others. It could be interpreted as an indicator that the description of aggressive behavior of other people causes the most negative emotions, which is shown in behavioral data of a linguistic task performance.

The effect of meditation consists in the general acceleration of performance of a linguistic task for all categories of emotional (but not neutral) sentences and in reduction of differences in the TPT between anxiety and aggression related sentences. This correlates with the assumption that meditation improves voluntary control over own emotions (Aftanas and Golosheykin, 2005; Marchand, 2012; Kasala et al., 2014). Our result could be interpreted as an indicator that meditators reduce the extent of negative perception of sentences about aggression that improves their control over execution of a grammatical task.

Other behavioral effects of meditation are connected with the alignment in AED for sentences about self and non-self. This effect happens due to improvement (in comparison with non-meditators) in accuracy in sentences about non-self with maintaining the accuracy for self. Differences in the TPT for the sentences about one-self and others in meditators remain the same, as well as in non-meditators. These results could be hypothetically interpreted based on data about cross-cultural differences in the neuronal processes underlying an assessment of self and non-self. It is known that in people who originate from individualistic (so-called “western”) cultures, a significantly stronger activation of the medial prefrontal cortex was associated when thinking about self rather than non-self, including when thinking about relatives (Kelley et al., 2002). In contrast to this, in people from collectivistic (“oriental”) cultures the thinking about self causes the same levels of activation of the prefrontal cortex as when promoted to think about relatives (Zhu et al., 2007; Wang et al., 2012). According to the self-report of our participants, the purpose of the Buddhist meditational practices is subjectively realized as “a dissolution of consciousness in the Universe.” As meditators aspire to reach this goal, the differences between themselves and others are perceived as an illusion that needs to be eliminated. It is possible to speculate that the lack of differences in accuracy of recognition of sentences about self and others in meditators is an indicator reflecting the effect of meditation on their consciousness. On the other hand, all of our participants (including non-meditators) consider the Buddhist community of Russia and Kazakhstan to belong to oriental, rather than to western culture. Moreover, the differences in TPT for sentences about self and non-self were still present in the meditators group. Another possible explanation for the effect of meditation is the general decrease in negative emotions that improves attention to those sentences which cause the most negative reaction in non-meditators. Such an assumption is in agreement with the results received when comparing reactions to anxiety and aggression related sentences.

Amplitude of frontal P300 peak is one of the most frequently noted neurophysiological markers of meditation (Cahn and Polich, 2009; Atchley et al., 2016; Telles et al., 2019). In the study of Vipassana meditation, it was revealed that dynamics of P300 together with theta and alpha spectral power differ in people with various durations of meditation practice (Kakumanu et al., 2019). Our ERP results showed the differences at the level of brain activity between meditators with long-term experience both from non-meditators and intermediate group of meditators. In long-term meditators the amplitude of P300 peak in all frontal and left temporal cortical regions was increased in comparison with other groups. At the same time, in all groups of participants higher amplitude of P300 in left frontal and temporal regions correlated with faster task solution for all categories of sentences. Frontal amplitude of the P300 peak is a well-known correlate of voluntary-controlled attention (Heinze et al., 1999). Our findings could be interpreted as the indicator reflecting the general improvement of ability to concentrate attention as a result of long-term meditation. It is known, that the right fronto-temporal areas are activated under processing of unconscious negative emotion (Satto and Aoki, 2006). Therefore, a decrease in the P300 amplitude in meditators with shorter experience can be hypothetically interpreted as an indicator of a general decrease of sensitivity to negative emotions.

The differences revealed in behavior may have several interpretations. Probably, it is a direct effect of meditation practice on self-assessment behavior. Another possible reason of revealed behavioral effects is differences in the personality traits of the participants. In our study, intergroup differences in the Big-Five factor markers were not identified. However, some of psychological properties, not covered by the Goldberg’s inventory, could exist between non-meditators and meditators before the latter began to practice meditation. These properties can have influence on recognition of self-related sentences.

It is necessary to notice that the differences in brain activity between the long-term meditators and other groups have no direct relation to the differences in the behavioral values. First of all, the behavioral differences have been found in the intermediate meditators as well, whereas the ERP differences were detected only in long-term meditators. Besides, ERP differences among the experimental groups did not concern the differences in reactions of the sentences about anxiety and aggression or about one-self and others, and were revealed for the reactions to all sentences, including the neutral ones. Respectively, in the present study we did not find the indicators of brain activity which are connected with the changes in perception of one-self and others, observed in behavioral assessment. On the contrary, our ERP findings reflect the long-term effect of meditation, which is shown at the perception of any speech-related information, including unemotional information about inanimate objects. A more detailed comparison of various behavioral and neurophysiological effects of meditation is a topic for our future research.

## Limitations

1. Although the groups of participants were balanced by sex and age to the best of our ability, inside of all groups there was significant age variability. The age range was between 20 and 66 years old: it was connected to age heterogeneity in groups of meditators. Besides, the researched samples were not few in number of participants. Our hypotheses about the effects of meditation on behavior and brain activity should be tested in larger groups.
2. Influence of inter-individual differences in the participants’ personality traits on the effect of meditation was not observed in this study and remains a subject for future investigations.
3. There were no direct relationships between intergroup differences in the recognition of self-assessment sentences with differences in amplitudes and latencies of ERP. ERP indicators correlated with the general time of a linguistic task performance for all categories of sentences and did not relate to the perception of sentences about one-self or others. Therefore, the relationship between behavioral and electrophysiological effects of meditation remains a topic of future research.
4. Non-meditators and meditators were tested during their engagement in the religious rites of the Lamaistic community. In a period of study execution, all participants lived in a special camp in temporary isolation from their usual living conditions. Possible influence of Buddhist rituals on the psychological state of non-meditators should be taken into account. This topic requires comparing all research participants with people not involved in the ritual practice of Buddhism, which is also a topic of further research.

## Conclusion

Meditative practice changes a perception of emotional coloring of written speech. Meditators have an increase in behavioral control of recognition of emotionally negative sentences about aggression, which is reflected in an increase in speed of performance of grammatical tasks. Meditation changes a perception of information about one-self and others. The differences in a recognition of sentences about one-self and others, which are characteristic of non-meditators, have not been observed in meditators. Effect of long-term meditation on brain activity is an increase in amplitude of P300 peak of ERP in frontal and left temporal cortical areas, which correlates with the reduction of the TPT. Revealed in this study is that ERP-effects of meditation are not specific to the emotional category of a sentence, and are not directly connected with processes of assessment of one-self or others.

## Data Availability Statement

All datasets generated for this study are included in the article.

## Ethics Statement

The study was approved by the Institute of Physiology and Basic Medicine ethics committee. All applicable subject protection guidelines and regulations were followed in the conduct of the research in accordance with the Declaration of Helsinki. The study aim was explained to all participants and they signed the informed consent.

## Author Contributions

ASav designed the study; organized scientific expedition to Baikal, participated in EEG data collection, undertook the statistical analysis, and wrote the first draft of the manuscript. ST and KS participated in EEG and behavioral data collection and analysis, and gave feedback to participants. YM is responsible for communication with the participants and leaders of the lamaistic Buddhism community of Russia, organization of scientific expedition, and control of ethical aspects of the study. ASap, SL, GKo and GKn participated in EEG and behavioral data analysis. All authors have read and approved the final manuscript.

## Conflict of Interest

The authors declare that the research was conducted in the absence of any commercial or financial relationships that could be construed as a potential conflict of interest.

## Footnotes

^1^http://www.geshe.ru/

^2^https://www.ncbi.nlm.nih.gov/pmc/articles/PMC3995046/
